# Exploring New Models for Implementing Sustainable Integrated Health Access for People in Vulnerable Positions: Protocol for a Mixed Methods Multiple Case Study

**DOI:** 10.2196/56197

**Published:** 2024-08-23

**Authors:** Sofie Buch Mejsner, Jane Aslaug, Mickael Bech, Viola Burau, Dorte Mark, Kathrine Vixø, Caroline Louise Westergaard, Michael Fehsenfeld

**Affiliations:** 1 Department of Public Health Aarhus University Aarhus Denmark; 2 Social, Health & Care Viborg Municipality Viborg Denmark; 3 Department of Political Science University of Southern Denmark Odense Denmark; 4 Central Regional Psychiatry Central Denmark Region Viborg Denmark; 5 The Danish Center for Social Science Research (VIVE) Copenhagen Denmark

**Keywords:** health care organization, social inequality in health, vulnerable people, integrated health access, healthcare access, accessibility, healthcare, Europe, social inequalities, health inequalities, mental illness, inequality, Denmark

## Abstract

**Background:**

Health care is a strongly universal right across European welfare states; however, social inequalities in health persist. This literature argues that health care organization is an important but overlooked determinant of social inequalities in health, as health systems buffer or amplify structural and individual health determinants. The Client-Centered Coordination Platform (3CP) model offers integrated health access to people with severe mental illness, through core groups of professionals from across health and social services.

**Objective:**

This study focuses on vulnerable people with severe mental health problems and aims to analyze how the model can give people with severe mental illness more integrated access to health and social care. This can form a stepping-stone for the upscaling of the 3CP model.

**Methods:**

We conduct a 5-year multiple case study of 3 municipalities in Denmark, where 3CP is being implemented. In a 1-year pilot study, we expect to gather quantitative registry data from the municipalities and the Central Denmark Region to explore the characteristics of people included in 3CP. We will also collect qualitative data, including 21 hours of observations; 36 interviews with users, professionals, and managers; and 3 focus groups across the 3 municipalities. In a subsequent, 4-year qualitative study, we aim to conduct 120 hours of observations, 120 interviews, and 24 focus groups. In parallel with the qualitative study, we will facilitate a cocreation process to develop tools for sustaining integrated health access.

**Results:**

As of January 2024, we have completed the individual interviews with users of 3CP and professionals and the focus groups. Individual interviews of managers will be conducted during the 1st quarter of 2024. The quantitative data are being collected.

**Conclusions:**

Inequality is one of the greatest challenges that European societies face. Understanding new and innovative approaches to integrated care may provide valuable solutions to the challenges posed. Especially understanding and designing health and social care systems that meet the needs and abilities of those users requiring them most, is vitally important to tackle inequality.

**International Registered Report Identifier (IRRID):**

DERR1-10.2196/56197

## Introduction

### Background

Health care is a strongly universal right across European welfare states: citizens have access to health care based on need rather than income. However, social inequalities in health persist. In Denmark, the risk of dying before the age of 65 years is almost 4 times higher for people who leave school after 9 years compared with those who continue with their education [1]. Social inequalities in health can be defined as systematic, avoidable, and unfair differences in health outcomes between social groups in a population [2].

The social inequalities in health are especially high among people in vulnerable situations, who experience many avoidable health and social care problems and a high risk of early death [3,4]. People in vulnerable situations are individuals who lack personal, material, and social resources to tackle the challenges of getting the right health and social care when in need [5]. They are a highly heterogeneous group and include people experiencing homelessness, people with disabilities, people with limited social support, and those with complex health conditions. Their life situations are highly complex and potentially volatile, and people in vulnerable situations typically have persistent contact with a wide range of different health and social care services [5-7]. Paradoxically, however, the chance to reach and obtain appropriate care in situations of perceived need remains poor [3,8].

Many countries struggle with increasing fragmentation of health and social care services including the Nordic countries [9]. Studies document the continued negative consequences of fragmentation for people in vulnerable situations, and it becomes evident that fragmented services affect this group particularly strongly [10].

Providing better access to health and social care for people in vulnerable situations should involve easy and flexible opportunities to reach and obtain an appropriate range of services [11]. Approaches to strengthen access to care have typically fallen into 2 camps, focusing on either an individual level (eg, empowerment, health literacy, social capital, trust, and expectations) or an organizational level to make services more accessible (eg, changes to service information, opening hours, location of services, outreach services, and appointment procedures) [6,9]. However, the reach of the second type of approach is often limited [12,13]. As Smithman et al [5] conclude, health care organizations must adapt their accessibility to vulnerable populations’ abilities, which requires innovations, tailored to reach and meet people in vulnerable situations’ specific needs (similarly Richard et al [6] and Hardin et al [12]). This calls for a new approach of integrated access that combines the individual level and organizational level approaches and adopts an intersectoral perspective across the health and social care system.

This is precisely the aim of the novel Client-Centered Coordination Platform (3CP) model that has been implemented in 3 municipalities of the Central Regional Psychiatry in Denmark. This model is inspired by Flexible Assertive Community Treatment (FACT) but emerged from the bottom-up and has a distinct intersectoral focus. The 3CP model combines an individual, needs-based approach with an intersectoral, integrated service organization. The 3CP requires specialized competence and organizational coordination which is costly, and 3CP is therefore targeted and limited to people who, in addition to severe mental illness, often have a limited level of social functioning, reflecting, for example, unstable housing, substance abuse, and financial problems [14]. The 3CP model includes an individually tailored core group of professionals from the regional specialist mental health services and the community-based services provided by municipalities. This multidisciplinary and cross-sectoral integration distinguishes 3CP from other, existing integrated care or case management models. The cross-sectoral implementation of 3CP in the Central Denmark Region, however, proved to be complex. In this study, we, therefore, investigate a cross-sectoral implementation of the 3CP model between the 3 municipalities and the specialist mental health services in the region.

There is not yet scientific evidence for the effectiveness of FACT in Denmark (which inspired the 3CP model). However, studies conducted elsewhere show positive results. Some main findings of FACT suggest increased remissions [15,16], fewer hospital admissions and inpatient bed use [17], better psychosocial functioning and more use of outpatient [18-20] care, increased compliance with treatment, decrease in unmet needs and improved quality of life [15], better working environments for professionals, and an increase in quality of care [21]. Many studies, however, often pertain to implementation, evaluation, or clinical issues and we need more knowledge on how vulnerable people with severe mental illness experience integrated access to care under this novel model; what specific organizational practices best support and sustain the integrated access to health and social care; and the distribution of health and social care costs in primary and secondary care. To answer these questions, this study adopts a theoretical perspective from the sociology of organizations.

### Objectives

The overall aim of this study is to analyze how 3CP can give people with severe mental illness more integrated access to health and social care. This can form a stepping-stone for the upscaling of the 3CP model to FACT.

More specifically, our aims are to (1) map out the characteristics of the people with severe mental illnesses participating in 3CP and their use of services; (2) explore how vulnerable people with severe mental illnesses perceive and engage in ongoing processes of integrated health and social care access, as well as treatment under the 3CP model; (3) analyze the organizational mechanisms of implementing and sustaining innovative practices for integrated health and social care access and ongoing treatment under the 3CP model; and (4) support people with severe mental illness, professionals, managers, and policy makers in sustaining practices for integrated care pathways and ongoing treatment.

## Methods

### Overview

**Figure 1:**
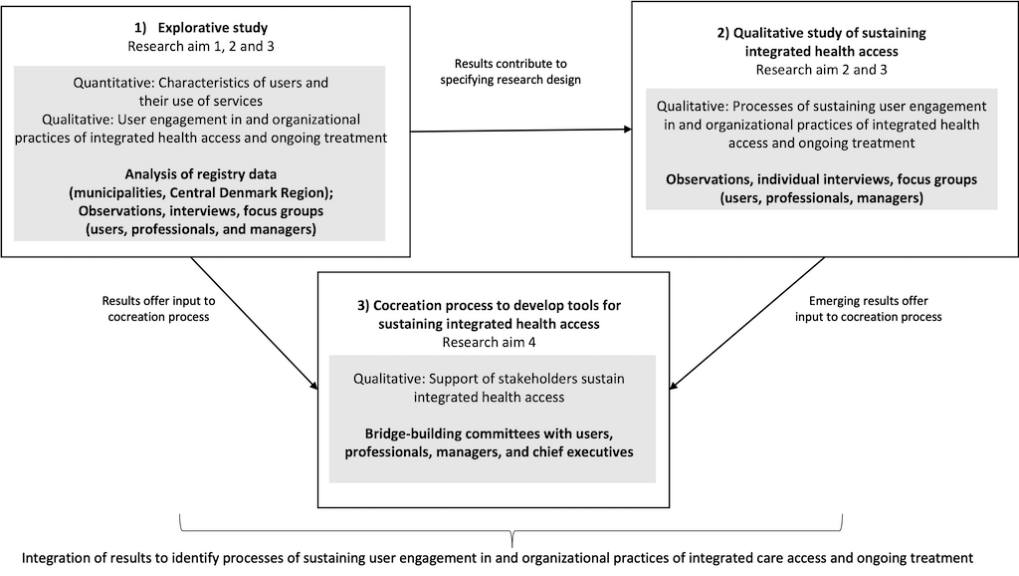
Overview of the study.

The investigation of complex and multilevel processes and systems requires a range of data. We conducted a 5-year multiple case study in Silkeborg, Skive, and Viborg municipalities in Denmark, where 3CP is implemented. This allows for comparisons across different local contexts and underlying organizational structures. The study includes three parts: (1) a 1-year explorative study of how 3CP can give vulnerable people more integrated access to care; (2) a 4-year qualitative study of how to sustain user engagement in and organizational practices of integrated health access and ongoing treatment under 3CP; and, in parallel, (3) a 4-year cocreation process to develop tools for sustaining integrated health access, through bridge-building committees (Figure 1).

### Theoretical Foundation

In many countries, mental health services are provided in a broad and complex structure between the health care and social care system. This coexists with the competing organizational and individual interests and pressures from, for example, regulators, decision makers, professionals, and the public. Navigating these structures may be challenging for people in vulnerable situations and there is, therefore, a need for organizations to meet the specific needs of individuals. This study uses the theory of inhabited institutionalism [22,23] as this allows examining both organizational structures and social interactions. The focus is on-the-ground activities of people doing things together against the background of how rules, norms, and culture operate in society [22,23]. This is because, these rules, norms, and cultures of health and social care organizations (institutions) shape and are shaped by the interactions of users, professionals, and managers. This approach is therefore ideally suited to understand the interplay between the institutions underpinning health and social care organizations, the processes of service delivery, and how this interplay shapes the unequal pathways of services for people [22,23].

### Ethical Considerations

This research will be carried out in accordance with the Helsinki Declaration. Ethical approval was waived by The Central Denmark Region Committees on Health Research Ethics (number 1-10-72-124-22). Participants gave signed informed consent for study activities.

The study involves people with severe mental illness, health and social care professionals, and managers. Health and social care professionals will assist in selecting people with severe mental illness to ensure that those involved have sufficient personal resources to participate. As the professionals possess a high degree of integrity toward the 3CP users and have gained their trust, professionals are highly suitable to select those who are able to participate in this research. The life course of the people with severe mental illness, who are included in 3CP core groups, varies (eg, from maintaining a stable daily life to having relapses). Therefore, the inclusion of participants will occur in a continuous dialogue between researchers, health and social care professionals, and managers. Data collection, handling, and analyses will further be done in accordance with national and European Union regulations, and participants will remain entirely anonymous. To ensure privacy, we do not use their names, initials, or other identifiers during the research process. All participants are given a number that identifies them. Participants in the study are also volunteers, receive information to make an informed decision about participation, and can withdraw at any time. Finally, a supervision team will be established as in-depth ethnographic encounters with people in vulnerable positions can pose distinct ethical challenges, that require self-reflection about the researcher’s position, biases, and relationship building [24,25]. This team of researchers, professionals, and users will discuss these issues as part of regular meetings.

### Research Setting

The 3CP model in this study distinguishes itself from the FACT approach in the following ways: there is less coordination across health and social care services, this occurs in a core group rather than a team with a shared caseload and joint management, meetings take place once a week instead of daily, and there are fewer professional groups involved and less flexibility in terms of scaling up or scaling down treatment.

The implemented model builds on a collaboration between mental health services in the Central Denmark Region, the municipalities of Skive, Silkeborg, and Viborg, and the Certification Centre for ACT and Flexible (F)ACT in the Netherlands. The research team consists of the Danish Center for Social Science Research, Aarhus University, and managers from the municipalities and the Central Denmark Region. This coproduction provides a unique platform for understanding the mechanisms and structures that are needed to be successful in implementing and sustaining models of integrated care access. It further safeguards the users of health and social care services, as the professionals act as advocates for this group.

### Danish Health and Social Care Setting

In Denmark, the responsibility for health and social care is shared among the national, regional, and municipal administrative levels. Each of the administrative levels has considerable autonomy in relation to the management, structure, and composition of services. Therefore, the contexts for the implementation and sustainment of the 3CP model can vary to some extent. The 3 included municipalities vary somewhat in size but possess a comparable population composition. At the same time, there are differences in the internal organizational structure and collaborative culture that may impact the implementation and sustainment of 3CP. For example, in each of the 3 municipalities, different departments are responsible for introducing 3CP and the extent of collaboration with the regional mental health services varies (Table 1). Great political and administrative autonomy is another reason why municipalities may implement and sustain 3CP differently.

**Table 1:** Local context of Client-Centered Coordination Platform core groups in Skive, Silkeborg, and Viborg.

Composition of core group			Organizational setting
**Skive**			Municipality: Centre for Mental Health and Substance Abuse
	Region: 3 nurses and 1 middle manager		
	Municipality: 9 outreach support workers and 1 social worker		
**Silkeborg**			Region or municipality: Integrated Mental Health Centre
	Region: 5 nurses, 2 doctors, 2 secretaries and 2 middle managers		
	Municipality: 4 outreach support workers, 3 substance abuse counselors, 2 secretaries, 3 middle managers, and 1 development officer		
**Viborg**			Municipality: Centre for Substance Abuse, Centre for Welfare Services
	Region: 8 nurses and 1 middle manager		
	Municipality: 4 outreach support workers and 1 middle manager		

### 3CP Model in Skive, Silkeborg, and Viborg

In Skive, Silkeborg, and Viborg municipalities, the 3CP core groups are organized differently, reflecting variations in resources (personnel and economic), values, and existing collaborations with the region (Table 1). In Skive and Viborg, the core group consists of housing support professionals from the municipality, a municipal manager (only in Skive), and specialist mental health professionals from the region. In contrast, in Silkeborg, professionals from the municipal center for substance abuse play a prominent role together with professionals from the specialist teams for attention-deficit/hyperactivity disorder and substance abuse in the region (Table 1).

However, the tasks of the three 3CP core groups are similar: following disease and symptom development, providing guidance and practical help in everyday life, working with rehabilitation, and supporting the person’s recovery. Those users included in 3CP teams experience severe mental illness and have chaotic life circumstances (eg, abuse, homelessness). The 3CP core groups thus provide long-term follow-up services for people living in their local community. The core groups offer services at two levels of treatment intensity: (1) regular individual follow-up in the core group and (2) intensive treatment and follow-up, where the user has contact with several group members, and where the core group discusses the user every week and plans what services are delivered by whom.

The core groups have shared caseloads and meet once a week to discuss those users who receive intensive treatment and follow-up support. Most users manage with regular individual follow-up, but if there is a risk of, for example, psychotic relapse, alcohol or drug relapse, or increased need for hospitalization, the core group intensifies its effort. The core group can provide this intensified effort in both the short and the longer term and during crises. When the crisis is over, professionals go back to normal individual follow-up.

### Data Collection (Parts 1 and 2)

#### Overview

All participants are chosen based on being part of a 3CP core group in 1 of the 3 municipalities. The users are together with the professionals from the core groups selected for individual interviews. This will be a dialogue between the researchers, health and social care professionals, and managers in the interdisciplinary core groups, to understand the users’ capabilities to participate. Second, the health and social care professionals, who are involved with the users, will also be invited to participate in individual interviews and focus group discussions. This will help provide an understanding of the 3CP users’ care pathways from their perspective and the perspective of the professionals surrounding them. The first 2 parts of the study involve various data collection methods, which are described below.

#### Explorative Study

In the 1-year explorative study, the collection of quantitative and qualitative data occurs in parallel (convergent mixed methods) to maximize synergies in the analysis of the data (Figure 1) [26]. This will offer a powerful platform for methodological triangulation [26,27]. Data consist of registry data and ethnographic material (eg, observations, focus groups, and interviews; Table 2). The explorative study focuses on mapping out the characteristics of people with severe mental illness included in 3CP core groups. On this basis, the study explores the experience and engagement of these people, and the health and social care professionals around them (eg, nurses, social workers, and substance abuse counselors). This study seeks to gain new knowledge about how people with severe mental illness navigate multiple encounters with health and social service providers, and their opportunities to reach and obtain appropriate access to services. It further focuses on the organizational practices across different local contexts that underpin processes of integrated access to care.

The quantitative study will present the demographic and socioeconomic profile of the people included in 3CP core groups, such as level of education, patterns of disease, labor market participation, and occurrence of homelessness. This study is based on registry data from the participating municipalities, the Central Denmark Region, and the national registries (eg, information on diagnosis, contacts with the health and social care services, and receipt of transfer payments; see Table 2). Data are collected in collaboration with the municipalities and the region, and data will cover a period of 5 years (2018-2022) and include 51 individuals included in 3CP core groups between 2020 and 2022. The characteristics of the individuals will be reported as simple yearly means. The qualitative data will include a total of 7 hours of observations of core group meetings or interactions between users and health and social care professionals, 12 interviews with users and professionals, and 1 focus group in each municipality (Table 2).

**Table 2:** Overview of planned data collection in Silkeborg, Skive, and Viborg municipalities.

Study			Characteristics
**Explorative study** **(1 year)**			
	Observations in each municipality		3 core group meetings and 2 professional or user interactions
	Individual interviews in each municipality		5 users of 3CP^a^, 5 professionals, and 2 managers
	Focus groups in each municipality		3-4 professionals
	In all municipalities		21 hours of observations, 36 interviews, and 3 focus groups
	**Registry data**		
		Silkeborg municipality	19 users of 3CP
		Skive municipality	16 users of 3CP
		Viborg municipality	16 users of 3CP
		All municipalities	51 users of 3CP
**Qualitative study** **(4 years)**			
	Observations in each municipality per year		3 core group meetings and 4 professional or user interactions
	Individual interviews in each municipality per year		2 users of 3CP, 4 professionals, 2 managers, and 2 chief executives
	Focus groups in each municipality per year		3 professionals and 3 managers or chief executives
	In all municipalities per year		120 hours of observation, 120 interviews, and 24 focus groups

^a^3CP: Client-Centered Coordination Platform.

#### Qualitative Study of Sustaining Integrated Health Access

This 4-year qualitative study aims to understand how to sustain user engagement in and organizational practices of integrated health access and ongoing treatment under 3CP in the longer term. Like in the explorative study, we will focus on both: how vulnerable people with severe mental illnesses perceive and engage in ongoing processes of integrated health and social care access as well as treatment; and how organizational practices by professionals, managers, and chief executives can sustain these innovative practices. In each municipality, we expect to gather the following material each year, over a period of 4 years: 10 hours of observations of core group meetings or interactions between users and health and social care professionals, 10 interviews with users, professionals, managers, and chief executives, and 2 focus groups with professionals and managers or chief executives, respectively. The interviews and focus groups will also be informed by the data collected in the explorative study (Figure 1).

### Cocreation Process to Develop Tools for Sustaining Integrated Care Access (Part 3)

To support the sustainment of integrated access to health and social care, this third part of the study consists of a cocreation process with local stakeholders. We will initiate and conduct bridge-building committees involving users of 3CP and the relevant professionals, managers, and politicians. Initially, a planning team with representatives from all 3 municipalities and the region will meet to discuss implementation. This is part of the cocreation process and will occur once a year and involve users, professionals, and managers. Subsequently, the planning team will meet once a year to share experiences of the bridgebuilding committee meetings (Table 3).

In each municipality, we aim to organize 5 bridgebuilding committee meetings of 2 hours each (over a period of 4 years). Each meeting will include 10 participants from across the municipality and the region; 2 users (who are able to participate), 4 professionals, 2 managers, and 2 politicians (Table 2).

The first meeting is a kick-off meeting, where the participants will assist in tailoring the structure and function of the meetings as part of a cocreation process. Next, there is a pilot meeting, before moving on to the regular committee meetings (Table 3). The final meeting focuses on sustainment to secure the long-term involvement of users, the adaptation of the 3CP model, and the continuous flexibility in care pathways. At all the committee meetings, the researchers involved in this study will develop questions and topics to facilitate discussion.

**Table 3:** Overview of bridge-building committees.

Years and its quarters		Committee meetings		
		Silkeborg	Skive	Viborg
**Year 1**				
	1	Planning team meeting	Planning team meeting	Planning team meeting
	2	#1 Cocreation workshop (kick-off)	#1 Cocreation workshop (kick-off)	#1 Cocreation workshop (kick-off)
	3	—^a^	—	—
	4	#2 Pilot	#2 Pilot	#2 Pilot
**Year 2**				
	1	Planning team meeting	Planning team meeting	Planning team meeting
	2	#3	#3	#3
	3	—	—	—
	4	—	—	—
**Year 3**				
	1	Planning team meeting	Planning team meeting	Planning team meeting
	2	#4	#4	#4
	3	—	—	—
	4	—	—	—
**Year 4**				
	1	Planning team meeting	Planning team meeting	Planning team meeting
	2	#5 Sustainment workshop	#5 Sustainment workshop	#5 Sustainment workshop
	3	—	—	—
	4	—	—	—

^a^Not applicable.

### Collecting Data With People in Vulnerable Positions

Ethnographic methods are characterized by flexibility and studies that are less fixed in terms of their specific form and extent of empirical material [28,29]. This is a highly suitable approach, as the users in our study are all vulnerable, as well as heterogeneous; they possess very different resources for participating in research. Their experiences with health and social care services may also change over time [29]. Likewise, the interactions between service users and service providers are “in the making” as our study progresses [28]. Even the organizational practices of 3CP are intended to be “flexible,” so that they can be adjusted to the needs of the user. We thus expect to combine various methods of data collection and do so in a flexible manner; this includes qualitative semistructured interviews, observations, photovoice [27,30], and informal conversations (see interview guide in Multimedia Appendix 1). Interviews will allow us to gain knowledge about the experiences of being part of a 3CP core group, as well as views of the organization of the services under the 3CP model. In relation to users, we will combine this with photo-voice to help us inquire into their underlying understandings of health and social care services. This method particularly provides a way of engaging people with intellectual disabilities, as their thoughts may be illustrated in a picture rather than expressed in wording [27,30]. We will provide the 3CP users with a disposable camera, where they can take pictures that they find relevant to their involvement in treatment. These pictures will be used in the interviews, to facilitate the discussion around their experiences with accessing health and social care services under the 3CP model. Observations and informal conversations will further give us more in-depth knowledge about their concrete experiences navigating the system as users and professionals, respectively.

## Results

As of January 2024, we have completed the individual interviews with users of 3CP and professionals and the focus groups (Table 2). We will, during February or March 2024, proceed with the individual interviews of managers in each of the 3 municipalities.

We are further in the process of collecting the quantitative data (Table 2).

## Discussion

### Principal Findings

Fragmented health and social care services affect people in vulnerable positions particularly strongly [4] and therefore organizations must adapt their accessibility to the needs and abilities of vulnerable populations. This study aims to analyze, if and how the 3CP model can give people in vulnerable positions more integrated access to care, drawing on an in-depth analysis of the interplay between individual needs and care-seeking practices, organizational practices, and underlying structures and mechanisms.

We anticipate that this study will contribute to existing knowledge in 3 respects. First, it will provide in-depth knowledge about pathways of health and social care seeking and the related barriers and facilitators, particularly under the 3CP model. Second, this knowledge can contribute to better understanding and addressing unequal access to health and social care. The causes of inequality in health are often assigned to “social determinants” (education, income, and gender) although these are correlations rather than causations; being a poor, low-educated man does not determine one’s health or access to health, even if more low educated men have poor health. The relationships between social factors and health are not simple and instead, this requires a more careful exploration of the “causes of the causes” [31]; that is, what are the specific links between social factors on the one hand and health on the other? Third, this study will contribute to existing knowledge on the relations between individual care practices and organizations’ practices of service delivery; in short, how organizations can facilitate and hamper access, especially across different sectors. This will contribute to addressing the lack of knowledge about the interplay between human interactions on the one hand and organizational structures on the other [32].

One limitation of our study is the uncertainty in collecting data with a group of highly vulnerable people. We therefore need to be flexible and adapt our strategies as we are in the field. Further, using the 3CP teams provides us with valuable knowledge on integrated health access, however, this is one context to be explored. Other models or settings might provide other insights.

### Dissemination of Results

To disseminate the results, we will reach out to stakeholders by presenting results on a regular basis at relevant workshops, conferences, and in (professional) journals. We will also deliver presentations at practitioner conferences on mental health care and substance abuse counselors such as those organized by the umbrella organization of Danish regions. The research team will publish the results of the study in at least 3 articles in international, peer-reviewed journals.

### Implications

The results of this study will strengthen integrated health access for vulnerable people. More specifically, the study will (1) identify strategies to help people in vulnerable positions with severe mental health problems to improve their experience of access across different sectors of the health and social care system; (2) develop a catalog of strategies for good practice for professionals and decision makers in municipalities, hospitals, and general practice to strengthen integrated access to care for people in vulnerable positions who experience severe mental health problems; and (3) offer a stepping stone for upscaling the 3CP model across other regions or municipalities and to other vulnerable groups, based on detailed knowledge about the specific contexts and mechanisms fostering integrated access to care.

### Conclusions

Inequality is one of the greatest challenges European societies face and there are many opportunities for future research within this field. However, understanding new and innovative approaches to integrated care may provide valuable solutions to the challenges posed. Especially, understanding and designing health and social care systems that meet the needs and abilities of those users requiring them most is vitally important to tackle inequality.

## Appendix

Multimedia Appendix 1 Interview guide.

## Abbreviations

3CP: Client-Centered Coordination Platform
FACT: Flexible Assertive Community Treatment
