# Supplementary material for: Exploring New Models for Implementing Sustainable Integrated Health Access for People in Vulnerable Positions: Protocol for a Mixed Methods Multiple Case Study
Source: JMIR Res Protoc. 2024 Aug 23;13:e56197. doi: 10.2196/56197 (PMC11380055; doi:10.2196/56197)
Supplement: Multimedia Appendix 1 [file resprot_v13i1e56197_app1.docx]

Annex 1 – Interviewguide

| **Analytical level** | **Themes** | **Suggested questions** | **Comments/notes** |
| --- | --- | --- | --- |
| Introductory questions | - Background knowledge - Access to what is immediately important for the informant to focus on - To create a safe and friendly situation | - "Please tell us a little about yourself and your background?" - Can you tell me about your life before you needed support? |  |
| Practice | - Understanding of health/disease - Experiences with healthcare services | Citizens:   - Why do you need support in your everyday life? - What does your disorder/disease mean to you? (e.g. in your everyday life) - When did you need support? - What do you need support for? - Are you also receiving treatment? (e.g. medical/admissions   - How long have you received help/support because of your disorder? |  |
| Practice & Relationships | Relationship between:   - Citizen – surrounding society - Citizen-professional - Professional-professional | Only citizens:   - Who do you get support from? (professional/close relationships/civil society etc)   Both citizens and professionals:   - How does it take place when you receive/give support (use a concrete example as a focal point - and go through the various situations) - How does it take place when you receive treatment? - What is important to you when you receive / give support.   Professionals only:   - Who do you talk to about the citizen? (ask both other professionals, close relations and civil society) - How does it take place? |  |
| Agency | - Ownership - Empowerment - ‘The citizen at the center’ - Starting point in the citizen's needs | Professionals:   - How do you understand the term recovery? - How do you see recovery in connection with your work in the core group? - Which professional benchmarks are the starting point for your help to the citizen? - Are there other benchmarks than professional ones? - Do you discuss these benchmarks with other practitioners? - In what situations? (describe concrete situations) - Do you discuss the landmarks with the citizen? (how does it take place?) - How (and from whom) do you get concrete information about what the citizen's needs are? - How is this knowledge included in your assessments? - How do you make decisions about what needs to happen? - Can you give examples of both individual and joint decisions and how they are taken?   Citizens:   - How is it decided what help / support you need? (and who decides that?) - How do you experience your relationship with the therapist in those situations? |  |
| Ruling relations | - How does 'contextual' conditions affect the work with 3CP | Professionals:   - How do you experience the organizational framework for working with 3CP?   - Economy   - Resources/personnel   - Communication   - Rules   - Quality standards - Has the framework changed since you started with 3CP?   - How?   Citizens:   - How do you experience the conditions/framework that you and the service provider have for working with your situation? |  |
